# Bacteriophage T7 DNA polymerase – sequenase

**DOI:** 10.3389/fmicb.2014.00181

**Published:** 2014-04-16

**Authors:** Bin Zhu

**Affiliations:** Department of Biological Chemistry and Molecular Pharmacology, Harvard Medical SchoolBoston, MA, USA

**Keywords:** bacteriophage T7, DNA polymerase, sequenase, DNA sequencing, marine phages

## Abstract

An ideal DNA polymerase for chain-terminating DNA sequencing should possess the following features: (1) incorporate dideoxy- and other modified nucleotides at an efficiency similar to that of the cognate deoxynucleotides; (2) high processivity; (3) high fidelity in the absence of proofreading/exonuclease activity; and (4) production of clear and uniform signals for detection. The DNA polymerase encoded by bacteriophage T7 is naturally endowed with or can be engineered to have all these characteristics. The chemically or genetically modified enzyme (Sequenase) expedited significantly the development of DNA sequencing technology. This article reviews the history of studies on T7 DNA polymerase with emphasis on the serial key steps leading to its use in DNA sequencing. Lessons from the study and development of T7 DNA polymerase have and will continue to enlighten the characterization of novel DNA polymerases from newly discovered microbes and their modification for use in biotechnology.

## INITIAL CHARACTERIZATION

DNA polymerases catalyze the synthesis of DNA, a pivot process in both living organisms and in biotechnology (Hamilton et al., 2001; Reha-Krantz, 2008). Family A DNA polymerases including *Escherichia coli* DNA polymerase I, Taq DNA polymerase, and T7 DNA polymerase have served as prototypes for biochemical and structural studies on DNA polymerases and have been widely used as molecular reagents (Patel et al., 2001; Loh and Loeb, 2005).

A DNA polymerase activity from bacteriophage T7 was first observed in an *E. coli* mutant deficient in DNA polymerase I infected with bacteriophage T7 (Grippo and Richardson, 1971). The initial characterization of T7 DNA polymerase was intriguing. Although the gene responsible for the polymerase activity was mapped to gene 5 (Hinkle and Richardson, 1974; Hori et al., 1979b), gene 5 protein (gp5) itself had what appeared to be no DNA polymerase activity but only ssDNA exonuclease activity (Hori et al., 1979a). Apparently a host component was required to reconstitute the full DNA polymerase (Modrich and Richardson, 1975a). This host factor turned out to be a small redox protein – *E. coli* thioredoxin (Modrich and Richardson, 1975b; Mark and Richardson, 1976). The redox capacity of thioredoxin, however, is not required for stimulation of the DNA polymerase activity (Huber et al., 1986). Instead thioredoxin plays a structural role in stabilizing the binding of gene 5 protein to a primer-template (Huber et al., 1987) and increase the processivity of the polymerase more than 100-fold (Tabor et al., 1987a), representing a unique function of this universal protein. Thioredoxin binds to a 71-residue loop of T7 gene 5 protein (Doublié et al., 1998), which is not present in other Pol I-type polymerases, resulting in a stable 1:1 complex (*K*_D_ = 5 nM; Tabor et al., 1987a).

Another intriguing finding during the initial characterization of T7 DNA polymerase is on its exonuclease activity. T7 DNA polymerase lacks the 5′–3′ exonuclease activity found in *E. coli* DNA polymerase I but does possess a strong 3′–5′ single and double stranded DNA exonuclease activity (Hori et al., 1979b). The double-stranded DNA exonuclease activity requires the presence of thioredoxin. Interestingly, various protein purification procedures, depending on the presence or absence of EDTA in the buffer, can generate T7 DNA polymerases that differ significantly in their exonuclease activity, resulting in two forms of DNA polymerase (Fischer and Hinkle, 1980; Engler et al., 1983). By comparison of the two forms of polymerase and careful tracking of the purification procedures, it was revealed that the exonuclease activity of T7 DNA polymerase could be specifically inactivated in an oxidation reaction by oxygen, a reducing agent and ferrous ion (Tabor and Richardson, 1987b). The easily modifiable exonuclease and extraordinary processivity of T7 DNA polymerase kindled the emergence of a powerful tool in the DNA sequencing era.

## SEQUENASE ERA

Invented by Sanger et al. (1977), the method of chain-terminating sequencing initiated a revolution toward the genome-sequencing era. However, the enzymes initially used for chain-terminating sequencing, the Klenow fragment of *E. coli* DNA polymerase I and avian myeloblastosis virus (AMV) reverse transcriptase, had low processivity (~15 nt for Klenow fragment and 200 for AMV reverse transcriptase, the latter has a relatively higher processivity but its rate of DNA synthesis is only several nucleotides per second). Processivity describes the number of nucleotides continuously incorporated by a DNA polymerase using the same primer-template without dissociation. Thus if the DNA polymerase used for chain-terminating sequencing is non-processive, artifactual bands will arise at positions corresponding to the nucleotide at which the polymerase dissociated. Frequent dissociation will create strong background that obscures the true DNA sequence. Although the issue can be partially improved by long time incubation with high concentration of substrates that may “chase” those artifactual bands up to higher molecular weight, this procedure is not an ideal solution since reinitiation of primer elongation at dissociation sites (usually regions of compact secondary structure or hairpins) is inefficient and may result in the incorporation of incorrect nucleotides. Although T7 DNA polymerase itself has a processivity of only a few nucleotides, the association with *E. coli* thioredoxin dramatically increases its processivity. Consequently, with T7 DNA polymerase termination of a sequencing reaction will occur only at positions where a chain-terminating agent (such as a dideoxynucleotide) is incorporated, yielding a long DNA sequence (Tabor and Richardson, 1987c).

A more severe problem with DNA polymerases used prior to T7 DNA polymerase is the discrimination against dideoxynucleotides, the chain-terminating nucleotides used in Sanger sequencing. Most of known DNA polymerases strongly discriminate against ddNTP. For example, T4 DNA polymerase, *E. coli* DNA polymerase I, Taq DNA polymerase, and Vent DNA polymerase incorporate a dideoxynucleoside monophosphate (ddNMP) at least a 1000 times slower than the corresponding deoxynucleoside monophosphate (dNMP). To use these polymerases in DNA sequencing a high ratio of ddNTP to dNTP must be used for efficient chain-termination. Even though the overall incorporation of ddNMP can be improved in such an uneconomic way, wide variation in the intensity of adjacent fragments still occur because the extent of discrimination varies with different DNA sequences and structures. T7 DNA polymerase, however, is at the other end of the spectrum, discriminating against ddNTP only several-fold. Thus a much lower concentration of ddNTP can be used with T7 DNA polymerase and the uniformity of DNA bands on the gel is much higher (Tabor and Richardson, 1987c). The discrimination was further lowered by replacing magnesium with manganese in the sequencing reaction (Tabor and Richardson, 1989a). With Mn^2^^+^ in an isocitrate buffer, T7 DNA polymerase incorporates dNMP and ddNMP at same rate, resulting in uniform terminations of sequencing reactions.

With the naturally endowed high processivity and the lack of discrimination against ddNTP, the only hindrance for T7 DNA polymerase as a DNA sequencing enzyme is its robust 3′–5′ exonuclease activity. Exonuclease activity increases the fidelity of DNA synthesis by excising newly synthesized bases incorrectly base-paired to the template. For applications like PCR it is often a desired feature. While for DNA sequencing such activity is detrimental since when the dNTP concentration falls, the rate of exonuclease activity increases close to that of polymerase activity, resulting in no net DNA synthesis or degradation of DNA. The associated exonuclease activity will also cause DNA polymerase to idle at regions with secondary structures in the template, producing variability in the intensity of signals. The iron-catalyzed oxidation mentioned above can produce modified T7 DNA polymerase with greatly reduced exonuclease activity, and this chemically modified enzyme was the basis for Sequenase and the first easy-to-use DNA sequencing kits commercialized by United States Biochemical Co. However, the residual exonuclease activity can still result in some loss of labeled DNA bands upon prolonged incubation (Tabor and Richardson, 1987b). Tabor and Richardson carried out an extensive chemical and mutagenesis screen for selective elimination of the exonuclease activity of T7 DNA polymerase. The rapid screen of a large number of mutants was based on the observation that exonuclease minus mutants of T7 DNA polymerase can synthesize through a specific hairpin region in the DNA template (Tabor and Richardson, 1989b). As a result many mutants deficient in exonuclease activity were revealed and among them a mutant lacking 28 amino acids in the N-terminal exonuclease domain had no detectable exonuclease activity, while its polymerase activity is significantly higher that of the wild-type protein. This mutant was the basis of version 2 of Sequenase. Sequenase pioneered development of themostable enzymes and facilitated the automation for high-throughput sequencing.

Degradation of a DNA fragment can occur via a nucleophilic attack on the 3′-terminal internucleotide linkage by H_2_O or pyrophosphate (PPi). The 3′–5′ exonuclease catalyzes the former reaction, generating dNMP or ddNMP. The latter reaction is called pyrophosphorolysis. As the reversal of polymerization, pyrophosphorolysis generates dNTP or ddNTP, sometimes resulting in “holes”: the disappearance of ddNMP labeled DNA fragments on the gel. By adding pyrophosphatase to the reaction to cleave PPi the pyrophosphorylysis can be eliminated (Tabor and Richardson, 1990). The combination of modified T7 DNA polymerase, manganese ion, and pyrophosphatase can generate accurate and uniform bands on a DNA sequencing gel to the extent that, the DNA sequence can be directly determined by the relative intensity of each band if different amount of the four ddNTPs are added at certain ratio (Tabor and Richardson, 1990).

Themostability is a highly desired feature for DNA polymerase. A thermostable enzyme like Taq DNA polymerase is superior for cycle sequencing, in which multiple rounds of DNA synthesis are carried out from the same template, with the newly synthesized DNA strand released after each cycle by heat denaturation. The heat stable DNA polymerase survives the denaturation step and is available for the next cycle of polymerization. Cycle sequencing allows much less DNA template and polymerase to be used in a sequencing reaction. In cycle sequencing low processivity is an advantage because a polymerase with low processivity cycles rapidly, decreasing the chance of strong specific stops. However, the strong discrimination against ddNTP (at lease 100-fold, often 10,000-fold) by most thermostable DNA polymerase was a significant obstacle for their use in cycle sequencing. Although the use of manganese ion can decrease the discrimination (Tabor and Richardson, 1989a), manganese has several disadvantages compared with magnesium such as narrow working concentration, precipitation, and less activity of DNA polymerase than that supported by magnesium ion.

Studies on T7 DNA polymerase led to one of the most elegant demonstrations of enzyme engineering and turned Taq DNA polymerase into “Thermo Sequenase.” To pursue the molecular mechanism underlying the discrepancy in discrimination against ddNTP among family A DNA polymerases, Tabor and Richardson swapped the five most conserved regions in the crevice responsible for binding DNA and NTPs between T7 DNA polymerase and *E. coli* DNA polymerase I (Tabor and Richardson, 1995), based on the 3D structure of *E. coli* DNA polymerase I. By an SDS-DNA activity assay, the “Helix O” from *E. coli* DNA polymerase I was observed to confer strong discrimination against ddNTP to T7 DNA polymerase. Further mutagenesis in this region revealed that the tyrosine-526 in T7 DNA polymerase or the homologous position phenylalanine-762 in *E. coli* DNA polymerase I was the single determinant for discrimination against ddNTP. When the corresponding residue, F667 in Taq DNA polymerase was replaced with tyrosine, the modified Taq DNA polymerase F667Y actually preferred ddNTP 2-fold over dNTP, comparing to the 6000-fold discrimination against ddNTP by the wide-type enzyme (Tabor and Richardson, 1995). Taq DNA polymerase F667Y, with its naturally endowed superior thermostability and engineered elimination of discrimination against ddNTP, was the basis for “Thermo Sequenase,” an enzyme that greatly expedited the Human Genome Project. The structure of T7 DNA polymerase in complex with a primed-template and a nucleoside triphosphate solved later (Doublié et al., 1998) revealed that the 3′-hydroxyl of the incoming nucleotide and the hydroxyl of Tyr 526 are both within hydrogen-bonding distance of the pro S_p_-oxygen of the β-phosphate and suggested that one or both of these interactions may be required for nucleotide incorporation. However, even with the structure one could not have predicted the dramatic effect of tyrosine-526 on nucleotide analog discrimination.

## AN IDEAL MODEL TO STUDY INTERACTIONS WITHIN A REPLISOME

T7 DNA polymerase consisting of T7 gene 5 protein and *E. coli* thioredoxin, together with T7 gp4 bifunctional primase-helicase, and gene 2.5 ssDNA-binding proteins constitute the simplest known replisome that mediates coordinated leading and lagging-strand DNA synthesis (Richardson, 1983; Debyse et al., 1994; Lee et al., 1998; Hamdan and Richardson, 2009). The concise organization of the T7 replisome makes it ideal for studies of the multiple interactions of DNA polymerase during the movement of the replisome such as loading of the polymerase (Zhang et al., 2011), polymerase exchange (Johnson et al., 2007), processive synthesis (Hamdan et al., 2007), and translesion synthesis (Zhu et al., 2011). Critical interactions for coordinated DNA synthesis including polymerase-thioredoxin (Johnson and Richardson, 2003; Ghosh et al., 2008; Akabayov et al., 2010; Tran et al., 2012), polymerase-helicase (Zhang et al., 2011; Kulczyk et al., 2012), polymerase-primase (Chowdhury et al., 2000; Zhu et al., 2010), and polymerase-gene 2.5 single-stranded DNA binding protein (He et al., 2003; Hamdan et al., 2005; Ghosh et al., 2009, 2010) interaction were extensively studied. The solid biochemical background of T7 DNA polymerase also attracted investigations using single-molecular methods (Lee et al., 2006; Hamdan et al., 2009; Pandey et al., 2009; Etson et al., 2010; Loparo et al., 2011; Geertsema et al., 2014).

## NOVEL T7-LIKE DNA POLYMERASES

DNA polymerases from microbes advanced DNA sequencing technology that in turn unveiled a much larger, diverse and unexplored microbial world. Metagenomics data indicates that the marine phages are the most abundant and diverse organisms on the earth (Suttle, 2005), of which 60–80% potential gene products do not match any in the database. A large portion of these gene products must be involved in the nucleic acid metabolism, thus one can expect numerous novel nucleic acid enzymes that can enrich the present toolbox of enzymes derived from a small group of characterized microbes. Indeed, our own initial effort on the characterization of marine phage polymerases have revealed unique features of a single-subunit RNA polymerase from marine cyanophage Syn5 that can complement the predominantly used T7 RNA polymerase for *in vitro* RNA synthesis (Zhu et al., 2013a, b). Characterization of marine phage DNA polymerases appears more promising since one can easily target numerous interesting DNA polymerases from the reported marine phage genomes, even just for T7-like or family A DNA polymerases such as those from cyanophage Syn5 (Pope et al., 2007) and P-SSP7 (Sullivan et al., 2005), phages infecting SAR116-clade bacterium (Kang et al., 2013) and marine ssDNA phages (Schmidt et al., 2014). Considering the high probability that the 60–80% unmatched genes may harbor novel polymerase genes, the marine phage is an unlimited treasure to contribute new polymerase tools that can fulfill niches in biotech industry. Characterization and engineering of T7 DNA polymerase has shown the value of identifying novel properties of nucleic acid enzymes.

## Conflict of Interest Statement

The author declares that the research was conducted in the absence of any commercial or financial relationships that could be construed as a potential conflict of interest.
